# Structural Features of Heparan Sulfate from Multiple Osteochondromas and Chondrosarcomas

**DOI:** 10.3390/molecules23123277

**Published:** 2018-12-11

**Authors:** Noemi Veraldi, Alessandro Parra, Elena Urso, Cesare Cosentino, Manuela Locatelli, Serena Corsini, Elena Pedrini, Annamaria Naggi, Antonella Bisio, Luca Sangiorgi

**Affiliations:** 1 Istituto di Ricerche Chimiche e Biochimiche G. Ronzoni, V. G. Colombo 81, 20133 Milan, Italy; noemi.veraldi@gmail.com (N.V.); urso@ronzoni.it (E.U.); cosentino@ronzoni.it (C.C.); naggi@ronzoni.it (A.N.); 2 IRCCS—Istituto Ortopedico Rizzoli, V. di Barbiano 1/10, 40136 Bologna, Italy; alessandro.parra@ior.it; 3 Department of Medical Genetics and Rare Orthopaedic Diseases—IRCCS, Istituto Ortopedico Rizzoli, V. di Barbiano 1/10, 40136 Bologna, Italy; manuela.locatelli@ior.it (M.L.); elena.pedrini@ior.it (E.P.); 4 Department of Medical Genetics and Rare Orthopaedic Diseases & CLIBI Laboratory—IRCCS, Istituto Ortopedico Rizzoli, V. di Barbiano 1/10, 40136 Bologna, Italy; luca.sangiorgi@ior.it

**Keywords:** heparan sulfate, multiple osteochondromas (MO), peripheral chondrosarcoma, human cartilage, NMR, HPLC–MS, EXT

## Abstract

Multiple osteochondromas (MO) is a hereditary disorder associated with benign cartilaginous tumors, known to be characterized by absence or highly reduced amount of heparan sulfate (HS) in the extracellular matrix of growth plate cartilage, which alters proper signaling networks leading to improper bone growth. Although recent studies demonstrated accumulation of HS in the cytoplasm of MO chondrocytes, nothing is known on the structural alterations which prevent HS from undergoing its physiologic pathway. In this work, osteochondroma (OC), peripheral chondrosarcoma, and healthy cartilaginous human samples were processed following a procedure previously set up to structurally characterize and compare HS from pathologic and physiologic conditions, and to examine the phenotypic differences that arise in the presence of either exostosin 1 or 2 (*EXT1* or *EXT2*) mutations. Our data suggest that HS chains from OCs are prevalently below 10 kDa and slightly more sulfated than healthy ones, whereas HS chains from peripheral chondrosarcomas (PCSs) are mostly higher than 10 kDa and remarkably more sulfated than all the other samples. Although deeper investigation is still necessary, the approach here applied pointed out, for the first time, structural differences among OC, PCS, and healthy HS chains extracted from human cartilaginous excisions, and could help in understanding how the structural features of HS are modulated in the presence of pathological situations also involving different tissues.

## 1. Introduction

Heparan sulfate proteoglycans (HSPGs) are amongst the most biologically important glycoconjugates, playing an essential role in a variety of events at the molecular level [1]. Their expression and structural characteristics vary depending on tissue and cell type, and they undergo dramatic changes during development and in disease processes [2,3,4]. Heparan sulfate (HS) biosynthesis initiates in the lumen of the Golgi apparatus with the formation of a tetrasaccharide linkage region (LR) which is conjugated to a proteoglycan core protein. Chain elongation occurs by alternating addition of *N*-acetyl-d-glucosamine (GlcNAc) and d-glucuronic acid (GlcA), resulting in 1→4 linked linear polysaccharide chains that vary in length from 20 to 80 units [5]. Exostosin-1 (EXT1, MIM608177) and exostosin-2 (EXT2, MIM608210) genes play a key role in HSPG biosynthesis, as they encode for two ubiquitously expressed transmembrane Golgi glycosyltransferases, EXT1 and EXT2, which catalyze HS polymerization forming a heterodimeric complex [6,7,8,9,10]. Immediately after the polymerization, an important modification process follows, involving the sequential action of a series of enzymes: *N*-deacetylase-*N*-sulfotransferase (NDST), which removes the *N*-acetyl group from GlcNAc by simultaneously adding a sulfate group (GlcNS); C-5 epimerase converting some GlcA residues into l-iduronic acid (IdoA); and various *O*-sulfotransferases, catalyzing the addition of sulfate groups in position 2 of IdoA or in positions 3 (rarely) and 6 of glucosamine [5]. A pivotal role in HS biological function is played by the sulfation of the 6-*O* position of glucosamine, which occurs through a combination of three intracellular HS 6-*O*-sulfotransferases (HS6ST1-3) and at least two extracellular endosulfatases (SULF1 and SULF2) [11]. These latter enzymes act after extracellular translocation of the glycosaminoglycan (GAG) chains, by cleaving off 6-*O*-sulfate groups in the internal part of the polysaccharide chain.

In developing growth plates, genetic approaches revealed that HSPGs, such as perlecan, syndecans, and glypicans, are essential players in many signaling pathways; however, with the exception of fibroblast growth factor 2 (FGF-2) signaling, the basis of their molecular interactions with specific molecules is yet to be fully elucidated [12,13]. HSPGs are involved in growth factors and morphogen diffusion, as well as in gradient formation and stabilization during cartilage development and skeletal growth [14]. They also affect signaling by hedgehog proteins (including Indian hedgehog, Ihh), bone morphogenetic proteins (BMPs), and members of the Wingless (Wnt) family of growth factors in a still undefined manner [15]. Moreover, HS is a potent inhibitor of remodeling activities present in bone and cartilage [16], and subtle changes in HS expression in this location could have profound effects on chondrocyte growth and/or differentiation. Mutations in EXT1 and EXT2 were linked to the development of multiple osteochondromas (MO). Previously called hereditary multiple exostoses (HME; MIM 133700 and 133701), MO is an autosomal dominant disorder with an incidence of 1/50,000 characterized by the occurrence of multiple benign cartilage-capped tumors that are typically located at the juxta-epiphyseal regions of long bones and often associated with skeletal deformities, functional limitations, and disproportionately short stature [17]. In addition, osteochondromas may occur at other sites, such as ribs, shoulder blade (scapula), and pelvis. Lesions in patients with MO were described in almost every bone formed by endochondral ossification; excluding some skeletal sites as calvaria, the most common locations are long bones (e.g., distal femur and proximal tibia).

Reduction or lack of HSPGs in MO may alter proper growth factor signaling leading to the aberrant bony growths. Bovèe et al. [18] showed that, in human osteochondromas, parathyroid hormone-related protein (Ihh/PTHrP) and FGF signaling are affected equally, and often simultaneously. A local perturbation in the Ihh diffusion and release from negative feedback control could cause premature chondrocyte differentiation, apoptosis, and ossification in the neighboring population [19].

For most MO patients, osteochondromas remains benign through life; however, in approximately 1–5% of cases, they progress to malignant tumors, known as secondary peripheral chondrosarcoma (PCS). To date, for MO patients, the high risk of developing PCS and the poor knowledge of the molecular mechanism underlying this process make regular radiological screening (e.g., X-rays) through the lifespan the only recommendation as a preventive strategy. Moreover, from a biological point of view, the effect of EXT1/2 mutations is yet to be clearly elucidated, due to the absence of a complete structural and molecular analysis of HS chains, whose polymerization—as already mentioned—is catalyzed by EXT genes. Knowledge of the variations in composition and organization of HS is, therefore, becoming increasingly important to clarify the relationship between structure and function in both benign and malignant lesions in MO disease. Many other hypotheses are present in literature on the enzymatic efficiency of the aberrant forms of EXT mutated gene products [20] and, in most cases, a hampered function is supposed, thus leading to the production of HS chains that differ from the wild-type synthesized ones [21,22]. Previous reports underlined a reduced amount of HS levels in MO-derived chondrocytes [23,24]; moreover, using immunohistochemical studies, Hameetman et al. [25] showed the absence of HS in the extracellular matrix (ECM) of cartilage affected by osteochondroma or chondrosarcoma (CS) and the accumulation of HS within the cytoplasm of MO chondrocytes. Nevertheless, the structural alterations that caused such an accumulation are unknown, and a real assessment of the biochemical status in MO cartilages—essential for developing new therapeutic strategies—is yet to be performed. In this context, potential prognostic/therapeutic targets—still lacking—are strongly needed, since MO is the most frequent skeletal dysplasia in the pediatric/adolescent age and severely affects the quality of life during patients’ lifespan.

The present work relates to the isolation of HS from healthy and pathologic cartilage excisions obtained from fetuses and prepubescent subjects and from patients affected by MO—considering benign OC or PCS, respectively—by applying a method previously set up [26]. The structural characterization of isolated HS was approached using mono and bidimensional NMR and especially using HPLC–MS analysis following exhaustive depolymerization with a cocktail of heparinases I, II, and III. The aim of the study was the identification of possible changes in structural features between healthy and pathological conditions, such as chain length, oligosaccharide composition, degree of sulfation, and a possible correlation with the original skeletal disorder. High-resolution mass spectrometry (HRMS) measurements played an essential role in this process, allowing detection and identification of HS species contained in such complex biological samples. Mass measurement accuracy combined with high speed and low-consuming sample characteristics make this analytical technique a major tool for distinguishing the correct component among all possible species exhibiting the same nominal mass. Furthermore, in some cases, mass fragmentation experiments were also applied to remove any doubt.

## 2. Results

### 2.1. Characterization of Cartilage Samples

Two groups of human cartilage excisions were analyzed for their HS content: healthy samples from fetal growth plate (FT) and prepubescent growth plate (GRP), and pathologic samples from multiple osteochondromas patients, including both osteochondromas (OC) and their malignant degeneration, chondrosarcomas (CS). All samples were freeze-dried after each step of purification and analyzed by NMR to verify the efficacy of the purification step and possibly the appearance of signals typical of HS. In Table 1, the list of samples is reported, together with the amount and area of cartilage explanted, age at resection, gender of patients, and the genotype characterization. As concerns fetal samples, no precise information was available on the cartilaginous area of excision due to the abortion procedure that did not allow proper recognition. The age of patients spanned from five to 29 years in the case of OC resection, and from 21 to 36 years in the case of CS, as expected since chondrosarcomas typically develop during adulthood, but earlier if compared to chondrosarcoma not derived from MO. All pathologic samples were from patients bearing mutations in *EXT* genes, both for OC (four in *EXT1* and four in *EXT2*) and malignant transformations, with a prevalence for *EXT1* gene in this last population (five in *EXT1* and one in *EXT2*). In all analyzed cases, the corresponding somatic variant was detected to be at homozygous status. Mutation details are shown in Table 1.

### 2.2. NMR Analysis of GAGs from Cartilage Samples

The major GAG components of cartilage tissue are chondroitinsulfate (ChS), hyaluronic acid (HA), and type II keratan sulfate (KS) linked to the proteoglycan aggrecan (see Supplementary Materials, Figure S1: Two-dimensional (2D) NMR spectrum of GAGs isolated from commercial aggrecan). GAGs were isolated from both fetal and prepubescent human cartilage and were treated with specific enzymes, such as chondroitinase ABC (ChABC) and hyaluronate lyase, to selectively eliminate ChS and HA. All samples were analyzed by ^1^H-NMR spectroscopy at each step of purification whereas heteronuclear single quantum coherence (HSQC) NMR was used to verify the disappearance of undesired species and to investigate the arising of typical HS signals. Fetal samples FT1–FT4, GRP1 and GRP2 were also subjected to digestion with keratanase, an endo-β-galactosidase, and keratanase II, which cleaves between a 6-*O*-sulfated glucosamine (GlcNAc6S) residue and galactose (Gal) or Gal6S, with the aim of completely degrading KS, followed by 3-kDa ultrafiltration to eliminate digestion products. GRP and pathologic samples were fractionated by ultrafiltration on 10-kDa cut-off membranes into two portions, retained fraction A (molecular weight (Mw) >10 kDa) and released fraction B (Mw < 10 kDa), with the aim of estimating the dimensions of isolated HS chains and to verify the possible differentiation of the three groups of samples (healthy prepubescent and pathologic MO and CS) at molecular weight distribution level. Actually, despite HSs being known from literature to have an Mw of at least 30 kDa [27], shorter HS chains were expected in our pathologic samples, thus explaining the use of small cut-off filters.

In healthy cartilage samples, the amount of residual ChS and KS chains that survived enzymatic degradation was so high—even after repeated steps—that both ^1^H-NMR and 2D-NMR analyses did not detect any signal from HS.

**Figure 1:**
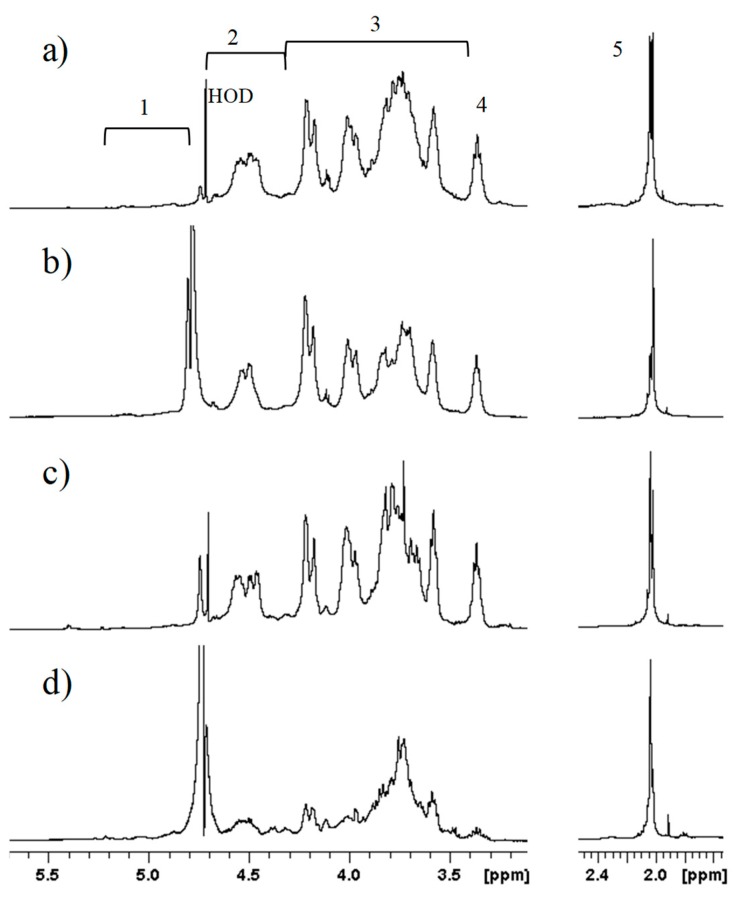
Superimposition of ^1^H-NMR spectra of glycosaminoglycan chains (GAGs) isolated from (**a**) chondrosarcomas sample 2 (CS2), (**b**) osteochondromas sample 1 (OC1), (**c**) prepubescent growth plate samole 1 (GRP1), and (**d**) fetal growth plate sample 3 (FT3). The main signal regions are indicated: 1, α-anomeric protons; 2, β-anomeric protons; 3, backbone signals; 4, d-glucuronic acid (GlcA) H2; 5, *N*-acetyl (CH_3_CO) of *N*-acetyl-d-glucosamine (GlcNAc). HOD = residual water.

**Figure 2:**
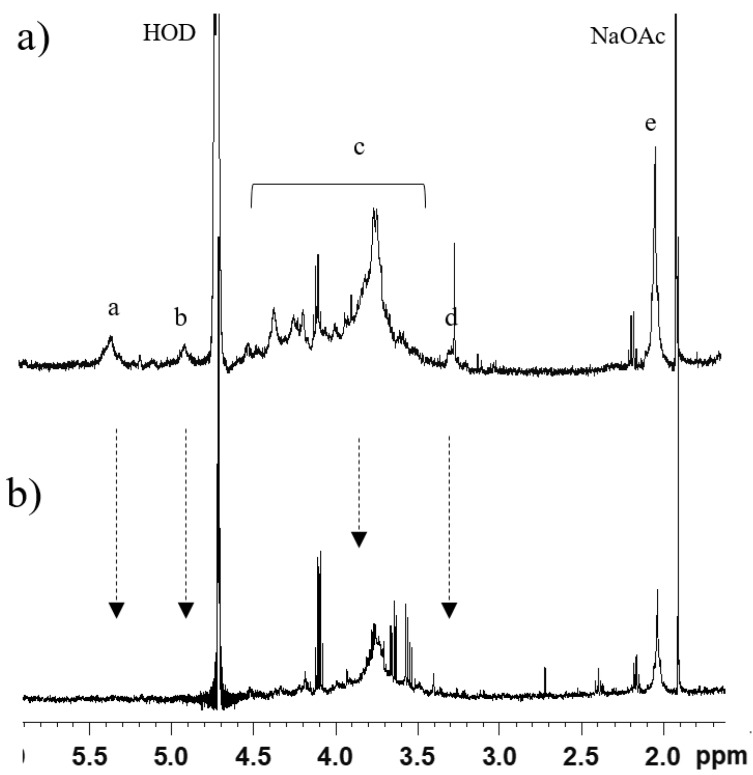
^1^H-NMR spectra of OC3-B (**a**) before and (**b**) after digestion with heparinases. Signals of heparan sulfate (HS) are indicated: a, H-1 of GlcNAc, *N*-sulfo-d-glucosamine (GlcNS), and 2-*O*-sulfo-l-iduronic acid (IdoA2S) (5.4 ppm); b, H-5 of IdoA (4.9–5 ppm); c, backbone signals; d, H-2 of GlcA and GlcNS (3.4 ppm); e, *N*-acetyl (CH_3_CO) of GlcNAc (2.0 ppm). Arrows indicate the decrease in signal.

GAGs isolated from osteochondromas and chondrosarcomas were subjected to the same enzymatic treatment and subsequent purification as healthy cartilage. Mono-dimensional ^1^H-NMR showed the presence of galactosaminoglycans in both healthy and pathologic samples (Figure 1). In particular, profiles were similar for OC1, CS2, and GRP1, which seemed to greatly differ from FT3. Deeper investigation using two-dimensional HSQC-NMR showed the presence of ChS and KS but not of HS in all samples, both healthy and pathologic, even after repeated digestion with hyaluronate lyase and ChABC (Supplementary Materials, Figure S2). Only a few samples among pathologic cartilage excisions were successfully cleared from ChS, and contained an amount of KS small enough to permit the detection of HS by ^1^H-NMR. HS was identified only in fraction B of OC3 (OC3-B, Figure 2) and CS5 (Supplementary Materials, Figure S3). In Figure 2a, the following HS signals were observable: *N*-acetyl group (CH_3_CO) of GlcNAc (2.0 ppm); H-2 of GlcA and GlcNS (3.2 ppm); H-5 of IdoA (4.9–5 ppm); and H-1 of GlcNS, GlcNAc, and IdoA2S (~5.4 ppm), the chemical shift of anomeric signals being due to the presence of calcium ions. Despite no further structural characterization being possible on this sample, the attribution of signals to HS was confirmed by their decrease or disappearance following digestion with heparinases (Hases) I, II, and III, and ultrafiltration, as indicated by arrows in Figure 2b.

^1^H-NMR spectra of healthy samples did not reveal any presence of HS, not even following the same enzymatic treatments as above (see Supplementary Materials, Figure S4: ^1^H-NMR spectrum of GRP2-B). Actually, the resistance to enzymatic digestion shown by ChS and type II KS hindered the purification of HS, preventing its detection or structural characterization by NMR in most cases. Accordingly, the approach adopted for structural analysis of isolated HS was the characterization of di/oligosaccharide composition using HPLC–MS following exhaustive enzymatic depolymerization by a cocktail of Hases I, II, and III [28].

### 2.3. HPLC–MS of HS Chains from Healthy Cartilage

All samples were subjected to digestion of HS with Hases, followed by desalting treatment and one-third of digestion products were analyzed by HPLC–MS. Inside each group (FT, GRP, OC, and CS), samples were analyzed in sequence, since only in this case could they be compared to each other regarding the intensity of peaks.

Products from fetal unfractionated HS were separated with a multistep gradient slightly different from the one used for all other samples in relation to the composition of the solvents used; therefore, oligosaccharides were eluted with a shorter retention time (RT) with respect to all the other samples analyzed so far. A commercial heparin sodium was also analyzed as reference standard to check chromatographic conditions and mass spectrometry signal response. LC–MS profiles are reported in Supplementary Materials, Figure S5.

Mass spectra information permitted the identification of oligosaccharide composition, in terms of chain length, number of sulfate and/or acetyl groups, and possible chemical modifications. In particular, the mass-to-charge ratio (*m*/*z*) allowed calculating the actual mass of each ion parent and formulating structure hypotheses. For convenience, the proposed structure was expressed using a code consisting of a sequence of three numbers (the numbers of monosaccharide residues, sulfate groups, and *N*-acetyl groups, respectively), preceded by a letter representing the non-reducing end residue. The symbol ΔU indicates that the first residue is a 4,5-unsaturated uronic acid (as expected by lyase action), or the symbol A for fragments starting with an amino-sugar residue, or U for saturated uronic acid.

**Figure 3:**
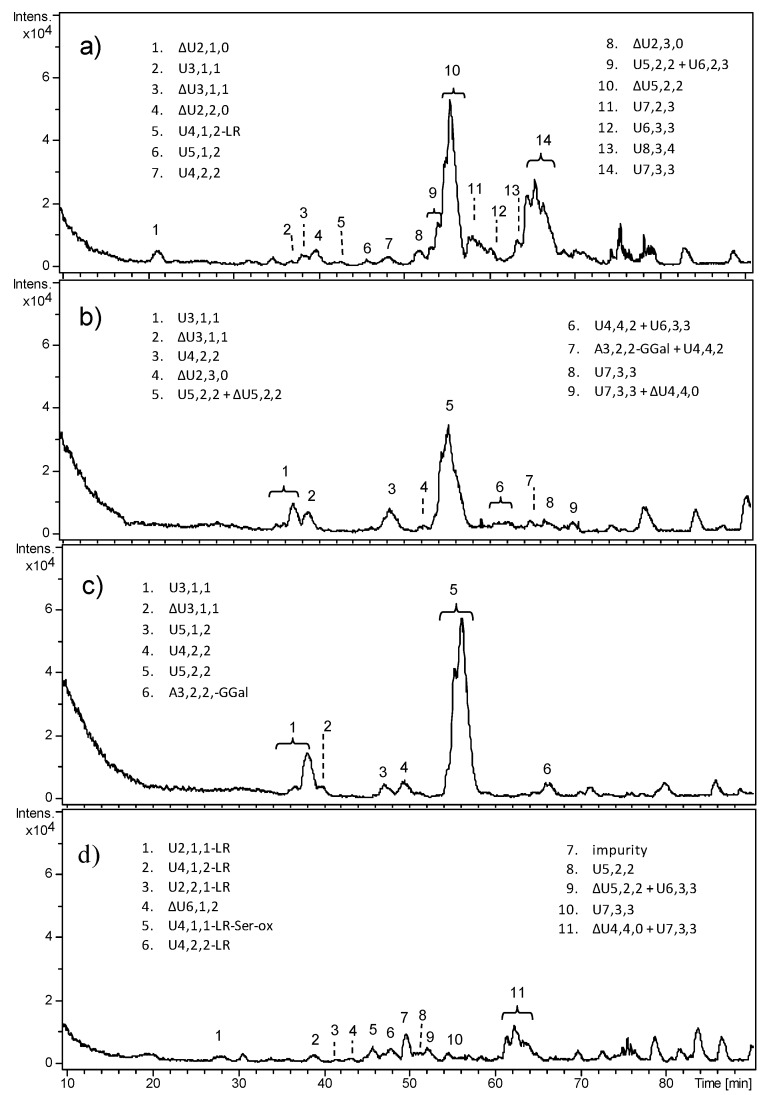
LC–MS profiles of heparinase digestion products from fetal HS: (**a**) FT1, (**b**) FT2, (**c**) FT3, and (**d**) FT4. Oligosaccharides were identified based on their mass/charge ratio (*m*/*z*) and labeled as follows: Δ indicates the unsaturated bond of the terminal uronic acid, followed by the number of monomers, sulfate groups, and acetyl groups. When a uronic acid (or a glucosamine) is present at both the reducing end (RE) and non-reducing end (NRE), it is indicated by U (or A). LR indicates the tetrasaccharide G-Gal_2_-Xyl of the linkage region. LR-Ser-ox indicates the presence of the linkage region bearing an oxidized serine residue.

All fetal samples revealed very complex and heterogeneous oligosaccharide profiles through HPLC–MS analyses (Figure 3), symptomatic of an incomplete digestion of HS, despite the enzyme/substrate ratio here employed being reported to produce a complete depolymerization of heparin to mostly disaccharides and a few tetrasaccharide and hexasaccharide species. Repeated digestion did not result in any further cleavage of the observed oligosaccharides. It is possible that ChS and KS surviving enzymatic degradation prevented the exhaustive digestion of Hases.

HPLC–MS profiles differed depending on the age of fetuses; FT2 and FT3 appeared as the most similar deriving from 17 weeks old fetuses, whereas F4 exhibited a few HS fragments. In FT1 and FT2, the predominance of the unsaturated pentasaccharide ΔU5,2,2 was observed, which includes two acetylated and monosulfated hexosamines. It could be attributable to fragments generated by ChS degradation that survived 3-kDa ultrafiltration.

Moreover, an unusual fragment that could not derive from lyases action, i.e., U5,2,2, was detected both in FT2 and FT3, here appearing as the major peak. It could be explained as a pentasaccharide sequence originally located at the non-reducing end of HS chain, released by the endogenous action of heparanase—an endo-β-glucuronidase which is known to hydrolyze HS chains leaving a glucuronic acid residue as reducing end (RE)—and not efficiently removed by 3-kDa ultrafiltration, as above. The same mechanism can be supposed also for the other odd sequences, e.g., U3,1,1 and U7,2,3.

However, beyond the overall complexity of HPLC–MS profiles, all the observed HS fragments exhibited a low degree of sulfation (less than one sulfate group per disaccharide (ds < 1)) and a high degree of *N*-acetylation. Moreover, the presence of a relatively high number of oligosaccharides not bearing an unsaturated uronic acid (as their *m*/*z* value was not consistent with the loss of a water molecule) resulting from the original non-reducing end (NRE) of HS chains, together with oligosaccharides bearing the tetrasaccharide linkage region (LR), would suggest the existence of original short HS chains.

**Figure 4:**
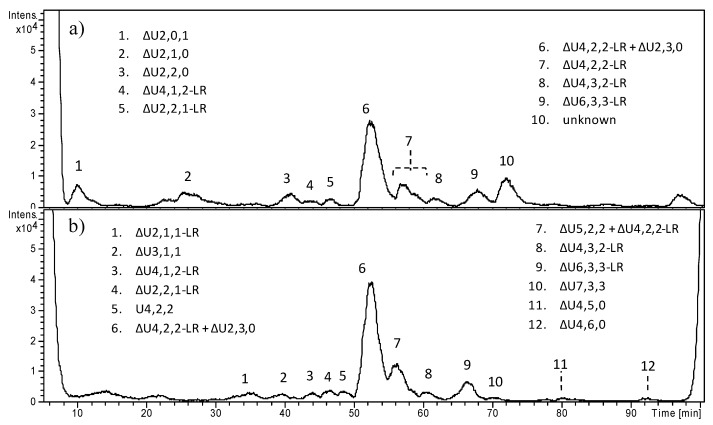
LC–MS profiles of heparinase digestion products from prepubescent healthy HS: (**a**) GRP2-A > 10 kDa, and (**b**) GRP2-B < 10 kDa. Oligosaccharides were identified by their mass/charge ratio (*m*/*z*) and labeled as follows: when a uronic acid (or a glucosamine) is present at both the RE and NRE, it is indicated by U (or A). LR indicates the tetrasaccharide linkage region G-Gal_2_-Xyl. Oligosaccharides for which an imprecise interpretation was found are labeled as “unknown”.

For prepubescent healthy HSs, the enzymatic cleavage by Hases I, II, and III turned out to be exhaustive. HPLC–MS profiles of fractions A and B of GRP2, reported in Figure 4, revealed the presence of several disaccharide species, also including the trisulfated ΔU2,3,0 together with tetra- and hexasaccharides, most of which bore the LR at their reducing end. The two fractions exhibited similar composition both from a quantitative and qualitative point of view. The number of original HS chains higher and lower than 10 kDa turned out to be comparable. Moreover, both highly sulfated and mixed *N*-acetylated/*N*-sulfated sequences were detected, such that the overall sulfation degree could be estimated as roughly close to 1.

**Figure 5:**
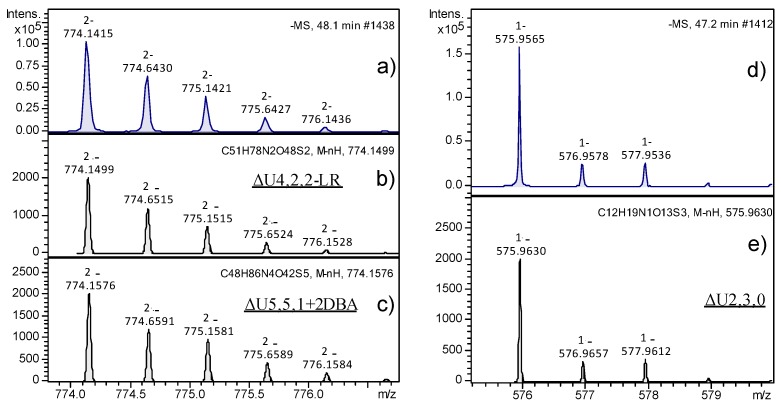
Mass spectra of selected oligosaccharides observed by ion-pair reversed-phase (IPRP) HPLC/electrospray ionization quadrupole time of flight (ESI-Q-TOF). On the left, the (**a**) experimental and (**b**,**c**) theoretical isotope patterns of a selected oligosaccharide together with the possible interpretations are reported. On the right, the (**d**) experimental and (**e**) theoretical isotope patterns of the standard trisulfated disaccharide are reported for comparison.

The peaks with the highest intensity observable in Figure 4 partially corresponded to *m*/*z* 774.14 (peaks 6 and 7 in fractions A and B) and *m*/*z* 546.0 ions (peak 7 in fraction B), and their assignment to specific structures was deeply investigated for a few reasons. With regards to the *m*/*z* 774.14 species, in principle, it could be attributed to two different structures, such as ΔU4,2,2-LR or ΔU5,5,1+2DBA, the latter corresponding to an adduct ion with two molecules of dibutylamine (DBA). In Figure 5, the experimental isotope pattern of the *m*/*z* 774.14 ion (panel a) was reported in comparison with the theoretical ones of the two candidate structures (panels b and c), showing their high similarity and the difficulty in correctly interpreting the mass spectra information. On the right (panels d and e of Figure 5), the isotope pattern distributions of the standard disaccharide ΔU2,3,0 showed a clear example of complete structure confirmation. The calculated error between the theoretical and experimental *m*/*z* ratio was between 10 and 20 ppm for both the interpretations compatible to the experimental mass signal; however, the smallest value was obtained for the chemical structure corresponding to ΔU4,2,2-LR. To unequivocally confirm such interpretation, MS/MS fragmentation of the 774.14 *m*/*z* ion was performed and fragments of the LR were revealed, thus confirming the initial hypothesis. Results are reported in Supplementary Materials, Table S1. With regards to the *m*/*z* 546.0 species, it was attributed to the pentasaccharide ΔU5,2,2, which appeared as an unusual HS digestion product by Hases I, II, and III, since the majority of resulting oligosaccharides were constituted by an even number of monomers. Nevertheless, the fragmentation results of the *m*/*z* 546.0 ion through the MS/MS experiment confirmed the hypothesized structure (Supplementary Materials, Table S2).

The main features of HS from healthy cartilage samples are summarized in Table 2, where an estimation of the ratio between fractions A and B—evaluated by considering the total area of LC–MS peaks of the corresponding fraction—and the trisulfated disaccharide (ΔU2,3,0) content, were reported. GRP samples differed for the chain length distribution: short HS chains (<10 kDa) turned out to prevail in GRP1, whereas, in GRP2, they were equivalent to long chains (>10 kDa). The two prepubescent GRP samples shared a very low or even undetectable content of ΔU2,3,0, and a similar degree of sulfation (≥1). In particular, the peak area of the trisulfated disaccharide ΔU2,3,0 (co-eluting with the linkage region fragment ΔU4,2,2-LR under the peak 6 in Figure 4), was calculated using an extracted ion chromatogram (EIC) at *m*/*z* 576.0, corresponding to its singly charged parent ion; then, its relative proportion was expressed as a percentage of the total area of all peaks detected.

With regards to fetal samples, for which the evaluation of chain length was not applicable, the content of highly sulfated sequences resulted negligible or even undetectable, and the sulfation degree appeared very low.

### 2.4. HPLC–MS of HS Chains from Pathologic Cartilage

Interestingly, HS was well detected in all pathologic samples, both OC and PCS, in most cases in a more consistent amount with respect to healthy samples. A picture of the main structural features of HS from pathologic cartilage is presented in Table 2.

With regards to OC cartilage, based on HPLC–MS profiles, a slight or significant prevailing presence of HS chains below 10 kDa appeared in seven samples, i.e., OC1–OC7, with a fraction A to B (Fr.A/Fr.B) ratio ranging from <0.01 to 0.9. Only for OC8 did longer HS chains result largely predominant (Fr.A/Fr.B = 3.1). Nevertheless, the most relevant and unexpected result was the consistent presence of highly sulfated sequences; the trisulfated disaccharide ΔU2,3,0, (i.e., derived from the IdoA2S–GlcNS,6S disaccharide) turned out to be the most important peak in six samples, i.e., OC1, OC3, OC4, OC6, OC7, and OC8. In addition, whereas, for most of pathologic samples, the amount of highly sulfated sequences in the two fractions A and B was quite similar, OC1 and OC2 exhibited much more ΔU2,3,0 content in Fr.B than in Fr.A: 85.5 vs. 31.4 and 72.6 vs. 12.4, respectively.

A number of fragments bearing the tetrasaccharide linkage region were also detected. The overall sulfation degree per disaccharide unit was roughly estimated over 2 for most OC, or slightly over 1 for OC2, OC5, and OC6.

**Figure 6:**
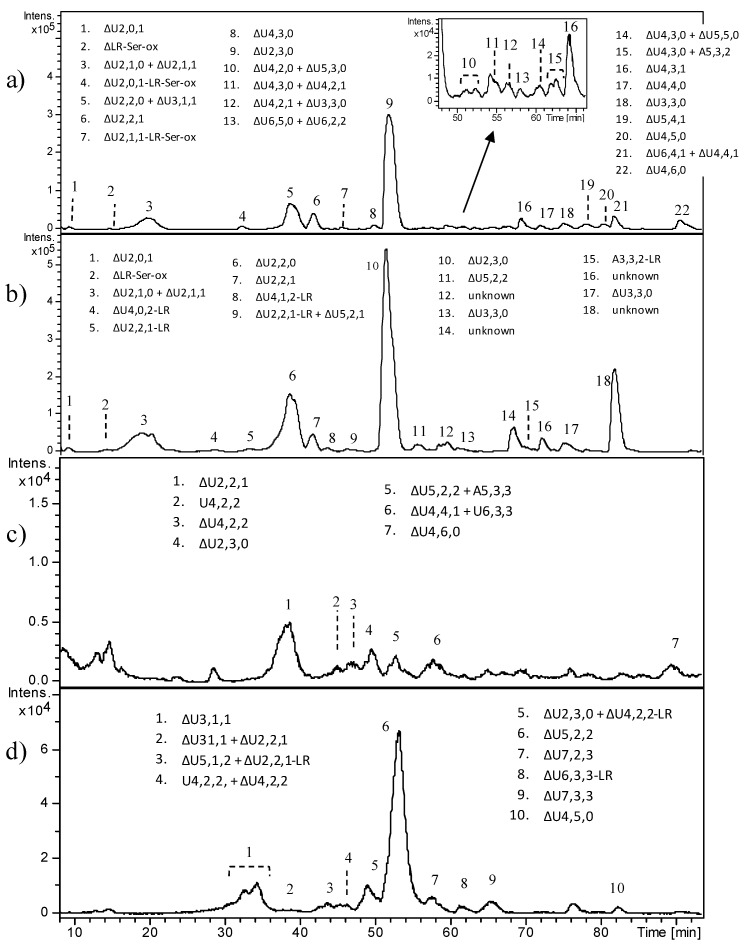
LC–MS profiles of heparinase digestion of HS from osteochondromas: (**a**) OC4-A, (**b**) OC4-B, (**c**) OC5-A, and (**d**) OC5-B. When a uronic acid (or a glucosamine) is present at both the RE and NRE, it is indicated by U (or A). LR indicates the tetrasaccharide G-Gal_2_-Xyl of the linkage region, while LR-Ser-ox indicates also the presence of the oxidized serine residue. Oligosaccharides for which an imprecise interpretation was found were labeled as “unknown”.

In Figure 6, the HPLC–MS profiles of HS digestion products obtained from OC4 and OC5 samples are reported, as examples of benign tumors carrying the deletion of *EXT1* and a severe mutation in *EXT2*, respectively. A number of peaks were detected especially in OC4, and their attribution is reported, ranging from di- to hexasaccharides (Fr.A). In both fractions of OC4, the trisulfated disaccharide ΔU2,3,0 clearly appeared as the predominant peak, accounting for 48% of all fragments in the whole sample. A different and less complex picture was shown by OC5, where the presence of less sulfated species together with the low amount of ΔU2,3,0 (10%) account for a moderate sulfation degree, apparently close to 1. In both samples, the presence of different species including the tetrasaccharide linkage region was detected. All other OC samples are reported in Supplementary Materials, Figures S6–S8.

In contrast with OC cartilage, HS from all CS samples resulted as prevalently composed of chains higher than 10 kDa. As shown in Table 2, the Fr.A/Fr.B ratio ranged from 1.5 to 12. As previously observed for the benign pathology, in five out of six CS samples, the percentage content of the trisulfated disaccharide was notable, varying from 52 to 67, allowing estimation of a sulfation degree higher than 2. CS1 and CS2, two excisions from the same tumors, appeared similar regarding the percentage of ΔU2,3,0 (67 vs. 53), but with a different proportion of Fr.A and Fr.B (4.6 vs. 1.5).

Furthermore, CS5 and CS6, a cancerous cartilage and its perichondrium, respectively, exhibited similar percentage content of highly sulfated sequences (58 vs. 52), but a different Fr.A/Fr.B ratio (6.3 vs. 3.2).

The highest number of long HS chains (Fr.A/Fr.B = 12) was detected in CS3, the only one of our series characterized by a mutation in *EXT2*, which also exhibited a huge percentage of ΔU2,3,0 (64). CS4, unlike all the others, displayed a 10-fold excess of short HS chains (Fr.A/Fr.B = 0.1), together with a content of trisulfated disaccharide and an overall sulfation degree comparable to healthy GRP. Nevertheless, a number of peaks attributable to GlcNS,6S was detected, suggesting that a possible anomalous hydrolysis occurred after the enzymatic heparinase digestion. Accordingly, this sample was not considered reliable for gathering information on pathologic HS features.

**Figure 7:**
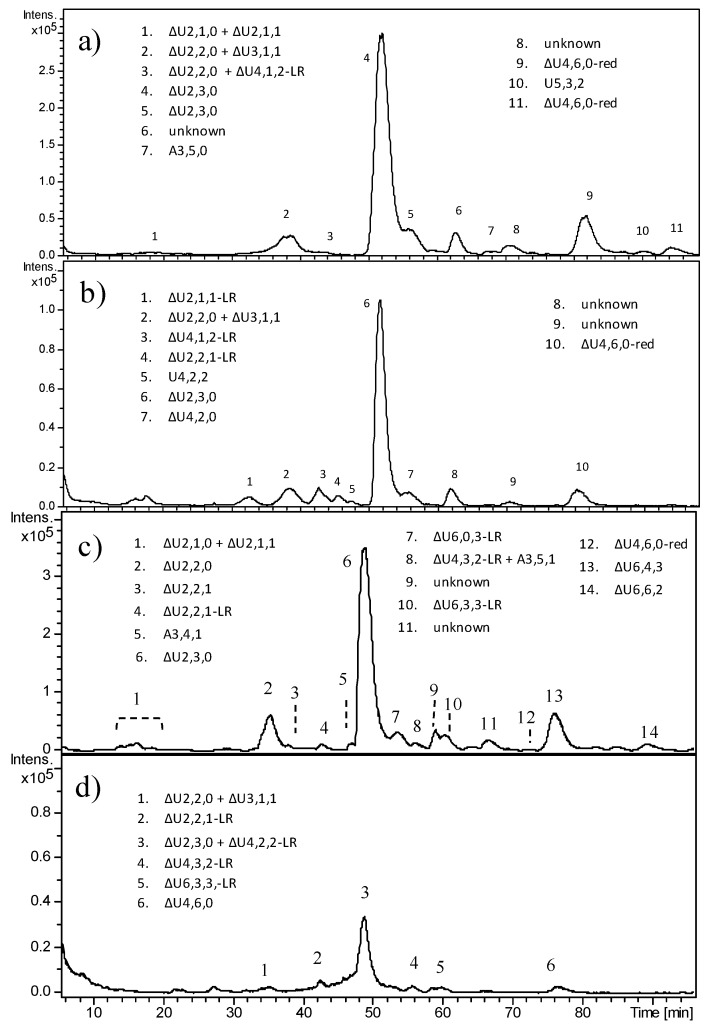
LC–MS profiles of heparinase digestion of HS from chondrosarcoma: (**a**) CS1-A, (**b**) CS1-B, (**c**) CS3-A, and (**d**) CS3-B. For some *m*/*z* ratios, more than one oligosaccharide structure was possible. When a uronic acid (or a glucosamine) is present at both the RE and NRE, it is indicated by U (or A). LR indicates the tetrasaccharide linkage region G-Gal_2_-Xyl. Oligosaccharides for which an imprecise interpretation was found were labeled as “unknown”.

In Figure 7, HPLC–MS profiles of the heparinase digestion products of HS from CS1 and CS3 are reported, as examples of malignant tumors carrying mutations in *EXT1* and *EXT2*, respectively. The high intensity of peaks corresponding to trisulfated disaccharide clearly appeared in both samples, their amounts corresponding to over 60% of the area of the total peaks. Ion species attributable to fragments containing the LR were detected in both samples.

An additional observation concerns the linkage region, i.e., the tetrasaccharide sequence connecting the HS chain to the protein chain of HSPG. In the HPLC–MS profiles of all healthy and pathologic samples, a different number of peaks attributable to fragments including the linkage region was detected, with a variable intensity depending on the chain length. The only exceptions were represented by CS4, once again, and both CS5 and CS6 (Supplementary Materials, Figures S9 and S10). Curiously, in these cases, the presence of *m*/*z* ions corresponding to the LR were not detected at all.

In the majority of pathologic samples, some peaks were labeled as “unknown” since their *m*/*z* values did not account for any known HS di/oligosaccharide. Most of these species were deeply investigated by MS/MS fragmentation experiments. Despite the different ions turning out to contain a variable number of sulfate groups, they were confirmed not to correspond to any regular or known (e.g., oxidized) HS-derived structure.

## 3. Discussion

Little is still known on how the disruption of genes, encoding for ubiquitously expressed enzymes, can cause a cartilage-specific disease like multiple osteochondromas.

Most MO patients carrying heterozygous loss-of-function mutations in *EXT1* or *EXT2* have reduced levels but not a complete lack of HS in their tissues, suggesting that, in tissues different from cartilage, the amount and/or structural characteristics of biosynthesized HSs are enough to support HS-mediated signalling [24]. This is in agreement also with the absence of osteochondromas at each cartilaginous growth plate site. In addition, Anower-E-Khuda et al. [29], investigating on the amount of HS in blood of MO patients, recently reported that heterozygous germline *EXT1* mutations are responsible for a partial systemic HS decrease of about 50% without affecting HS structure.

To date, a number of investigations evaluated the expression of different enzymes involved in HS chain elongation, or sulfation or extracellular sulfation remodeling using MO animal models or, alternatively, cell lines with specific deficiencies [30,31,32], thus inferring information about a possible HS composition in OC or PCS conditions.

Studies were also performed assessing the amount and/or structural features of HS produced by cultured osteochondroma chondrocytes or chondrosarcoma cell lines [24,32]. Despite it being well known that the HS structure is highly tissue- and environment-specific [33,34], direct structural studies on HS extracted from surgical retrieval specimens are yet to be reported.

The present paper relates to the structural characterization of HS from osteochondromas and chondrosarcomas and from healthy cartilage tissues, with the aim of identifying and understanding possible structural differences and diversities. At the beginning of this study, two major issues were considered: the lack of information in literature about the structure of cartilaginous HS, and the difficulty in obtaining healthy cartilage from human donors. For the present study, prepubescent growth plate and fetal cartilage samples were considered as healthy tissues. Osteochondromas possesses the equivalent biological behavior of a growth plate that ossifies and closes with the onset of skeletal maturity [35]; therefore, comparison between pathologic lesions and healthy cartilage should be done considering growth plates as normal counterparts of osteochondromas.

The isolation and analysis of human cartilaginous HS were approached as previously described by Parra et al. [26]; mono and bidimensional NMR and HPLC–MS after exhaustive and specific depolymerization of HS with Hases I, II, and III were applied for investigating structural features.

Nevertheless, due to the very small amount of HS found in cartilage GAG extracts with respect to the prevailing chondroitin sulfate and keratan sulfate components, NMR turned out to be ineffective for its structural investigation. Even after repeated specific enzymatic treatments and ultrafiltration steps for removing undesired GAG species, in most cases, HS signals were not detectable by ^1^H-NMR, even by bidimensional HSQC-NMR. Accordingly, in this study, HPLC–MS analysis of depolymerized products turned out to be the most eligible technique for profiling HS composition.

First of all, despite the interfering presence of residual ChS and KS, HS was detected in all extracts, and most pathologic samples surprisingly revealed a higher amount of HS relative to healthy growth plate cartilage. Moreover, by observing the overall results, two important HS structural features, i.e., sulfation degree and chain length, turned out to be affected in pathological situations. Most pathologic samples of both OC and CS exhibited a significantly higher sulfation degree with respect to healthy growth plate, mainly due to the consistent presence of the trisulfated disaccharide IdoA2S–GlcNS,6S. Highly sulfated HS chains could be the result of either an excess of intracellular sulfation activity, by NDST in particular, or a reduced or missing extracellular editing process by endosulfatases, or both. The relative amounts of NDST1, EXT1, and EXT2 determine the outcome of HS biosynthesis. The NDST enzymes are believed to play a key role in designing the sulfation pattern during biosynthesis because further modifications, i.e., C-5 epimerization and *O*-sulfations, occur mainly in *N*-sulfated regions. An increase in *N*-sulfation level was observed by Presto et al. [36] in EXT2/NDST1-overexpressing human embryonic kidney (HEK) cells; the polysaccharide synthesized in these cells actually resembled heparin.

In the absence of EXT1, NDST1 forms a complex with EXT2, resulting in increased *N*-sulfation levels of HS chains. Consistent with these observations, *N*-sulfation of HS chains increased in EXT1-deficient mouse fibroblasts gro2C cells, as shown by Okada et al. [31]. These data suggest that decreased expression of *EXT* genes could affect NDST activity and the final sulfation of HS chains.

On the other hand, aberrant distribution of HSPG was recently described in chondrosarcomas and osteochondromas in the presence of normal expression of *EXT* genes and in *EXT1* deletion, respectively [25,37]. Through histochemical studies, it was demonstrated that HSPGs were no longer present at the cell surface but accumulated in the cytoplasm, specifically in the Golgi apparatus. A similar event could further explain the high HS sulfation degree detected in our CS samples, as a result of missing action of extracellular endosulfatases SULF1 and SULF2. This last finding agrees also with the increased 6-*O*-sulfation level identified in HS from the grade III chondrosarcoma cell line (CCLIII) by Waaijer et al. [32]. They showed increased HS6ST1 and HS6ST2 expression during chondrosarcoma progression. As 6-*O*-sulfation plays an important role in signal transduction, a large amount of evidence [38,39,40] reported its potential role and implications in cancer; thus, an altered sulfotransferase expression might also be associated with chondrosarcoma progression [32].

With regards to chain length, the two groups of pathologic samples exhibited two opposite trends: most OC samples turned out to be comparable to healthy growth plate cartilage showing a prevalence of HS chains below 10 kDa, corresponding approximately to 18–20 disaccharide units, whereas CS samples exhibited a prevalence of longer chains, above 10 kDa. The prevalently short HS chains present in healthy and OC samples could be the result of heparanase (HPSE) hydrolysis as confirmed by the number of odd fragments found in our HPLC–MS profiles, compatible with its specific enzymatic action. HPSE is an endo-β-d-glucuronidase, prevalently located in the extracellular matrix, that cleaves the HS chains of HSPG yielding fragments of variable size, typically ranging from 10 to 20 sugars. Its activity was long detected in several cell types and tissues and, importantly, is often upregulated in human cancers [41,42]. HPSE was also detected and found to be widespread in the cartilaginous cap of osteochondromas, whereas, in normal growth plate cartilage, it appeared to be restricted to the hypertrophic zone and perichondrium [22,30,43]. On the other hand, the possible sequestration of HSPG in the Golgi apparatus discussed above could also explain the prevailing presence of long HS chains in CS samples, as they survived the hydrolytic action of HPSE.

Considering the *EXT* germline mutation, no correlation between the composition of HS chains and the type and/or the position of the variant along the genes could be observed, apparently revealing that any type of mutation will lead to an impairment in the EXT machinery; this is in accordance with what was observed in some genotype–phenotype correlation studies where the *EXT* mutation type/location did not affect the severity of the disease [44].

Only one OC sample, i.e., OC8, exhibited exceptional HS features with respect to the others, resulting as mainly composed of long HS chains, mimicking the behavior of CS samples. Such exceptional features can be explained by means of a clinical revision of the history of this patient. OC8 was correctly classified as an osteochondroma; also, the anatomo-pathological findings (i.e., the thickness of the cartilage cap) showed a borderline value of 9 mm, suggestive of a malignant transformation onset. Actually, the same patient developed PCS with a malignancy grade between 1 and 2 in the same site.

As concerns the HS extracted from the perichondrium of a chondrosarcoma, i.e., CS6, it turned out to have the same structure of HS from the correspondent hyaline cartilage; despite our analysis being limited to one sample, it would suggest that changes in HS are present in all zones of the cartilaginous tissue. HS allows the perichondrium to act as an anti-chondrogenic border around the growth plate [30]; thus, mutations in the *EXT* genes and, consequently, in HS structure and amount would lead to enhanced chondrogenic response of perichondrium.

Together with the relevant unusual content of highly sulfated HS sequences, in most pathologic samples, some other structural anomalies were revealed by LC–MS analysis, consisting of unknown sulfated oligosaccharide structures that did not turn out to correspond to any canonical or known HS-derived structure. This aspect deserves a future in-depth investigation.

A minor but not negligible result arising from the present study was the absence even of traces of the linkage region sequence in two CS samples: a chondrosarcoma with *EXT1* deletion and the corresponding perichondrium. This finding suggests the importance of also investigating the core protein of the proteoglycan, as its role in the HSPG function cannot be underestimated.

## 4. Materials and Methods

### 4.1. Human Cartilage Samples

Four fetal growth plate cartilage samples (FT1–FT4) were obtained courtesy of Dr. Salvatore Romeo and Dr. Angelo Paolo Dei Tos, Pathology Department of Treviso Hospital, Treviso, Italy. All other fresh-frozen tissue samples were obtained from patients who underwent surgery at Istituto Ortopedico Rizzoli (IOR, Bologna, Italy) and were immediately stored in liquid nitrogen after excision. Two prepubescent healthy growth plate cartilage samples (GRP1 and GRP2) were obtained from patients affected by diseases not related to MO. For each osteochondroma, microdissection of the cartilage cap was performed by the Pathology Department (IOR) and all histologic slides were reviewed by a pathologist to confirm the benign or malignant features. Eight tissue samples (OC1–OC8) were from seven patients affected by MO (OC6 and OC7 were from two different areas of the same resection). Six tissue samples (CS1–CS6) were from four patients affected by primary tumors MO-derived: CS1 and CS2 were from two different chondrosarcomas of the same lesion, whereas CS5 and CS6 were from different areas of the same lesion, with C6 being from the perichondrium. The amount of all samples was about 150–200 mg of wet tissue each, except for the GRP samples that were both about 100 mg of wet tissue. A commercial heparin sodium (Shenzhen Hepalink Pharmaceutical Co, Shenzhen, China) was used as a reference standard for LC–MS analysis.

For each pathologic tissue, the corresponding blood sample was also collected. Ethical approvals (ID 0021288/2013 and ID 0026288/2014) were obtained for each sample collection and subsequent analysis by the Ethic Committee of IOR on 26th of June 2013 and on the 25th of July 2014.

### 4.2. Identification of EXT Mutations

Determination of mutations was also carried out. DNA was extracted both from the blood samples and from a small part of the tissue, in the latter case, by means of digestion with proteinase K and separation of the genomic DNA onto a silica membrane mini spin column (DNeasy Blood & Tissue kit, Qiagen GmbH, Hilden, Germany). DNA quality was checked with a Nano Quant Infinite M200 instrument (Tecan Group Ltd., Mannedorf, Switzerland) before analyses. Complete mutational screening of EXT1/EXT2 coding regions and exon–intron junctions was performed using primer pairs and PCR conditions previously described [44]. Starting from the genomic DNA, subsequent pre-screening analysis was performed by denaturing high-performance liquid chromatography (dHPLC, WAVE System Model 3500HT, Transgenomic, Omaha, NE, USA) followed by Sanger sequencing of samples with an abnormal dHPLC elution profile (ABI PRISM 3130XL, Applied Biosystems, Foster City, CA, USA); in the case of negative results, multiplex ligation-dependent probe amplification (MLPA) with an MRC-Holland kit (SALSA MLPA P215 EXT probemix + SALSA MLPA EK1 REAGENT KIT) as performed to detect copy number variations according to manufacturer’s instructions, as previously described [45]. When the *EXT* heterozygous causative mutation was identified, its presence was also confirmed in the somatic DNA.

### 4.3. Isolation of GAGs

A published work-up procedure was used for the extraction of GAGs from human cartilage [26]. Briefly, GAGs were isolated by proteolitic cleavage with Proteinase K (Sigma, St. Louis, MO, USA) and DNase I (Sigma) treatment in piperazine-*N*,*N*′-bis(2-ethanesulfonic acid) (PIPES) buffer followed by ultrafiltration (healthy samples, molecular weight cut-off (MWCO) 3 kDa, Spectrum Labs, Rancho Dominguez, CA, USA) or dialysis (MWCO 0.5–1 kDa, Float-A-Lyzer, Spectrum Labs, Rancho Dominguez, CA, USA) to remove digestion fragments. In order to eliminate PIPES, pathologic samples were loaded onto a QAE-sephadex column and GAGs were eluted with 0–2.5 M NaCl, while PIPES was eluted with 3 M NaCl. Collected fractions were desalted by overnight dialysis (0.5–1 kDa cut-off) followed by gel permeation (TSK HWS40, Toyopearl, Tosoh Bioscience GmbH, Griesheim, Germany), and analyzed by NMR.

### 4.4. Purification of Samples by Enzymatic Digestion

Purification was carried out by specific enzymatic degradation of undesired GAG components, i.e., ChS and KS. Digestion of chondroitin sulfates by chondroitinase ABC (Sigma Aldrich, St. Louis, MO, USA) was carried out in 50 mM phosphate buffer and 50 mM sodium acetate (1:1 *v*/*v*), pH 8 at 37 °C for 48 h under continuous dialysis with a SpectraPor Float A Lyzer, MWCO 500–1000 Da (Spectrum Medical Industries, Inc., Rancho Dominguez, CA, USA) against 50 mM phosphate buffer and 50 mM sodium acetate (1:1 *v*/*v*). Each sample was further treated with hyaluronate lyase (Sigma Aldrich) in 50 mM sodium acetate and 10 mM calcium chloride, pH 6 at 37 °C for 48 h and subjected to ultrafiltration onto Amicon ULTRA centrifugal filter units (MWCO 3 kDa) to reduce the salt concentration. Keratanase and endo-β-*N*-acetyl-glucosaminidase digestions were performed in sodium acetate pH 4.6 at 37 °C for 48 h.

### 4.5. Fractionation by Ultrafiltration Through MWCO 10-kDa Filter

Following all degradative enzymatic treatments, samples were fractionated by Amicon ULTRA centrifugal filter units (MWCO 10 kDa) into two fractions: over 10 kDa (A) and under 10 kDa (B). Due to the scarce amount of extracts obtained from fetal cartilage samples, fractions 1–4 did not undergo such a treatment.

### 4.6. NMR Characterization

Samples were dissolved in 1 mL of D_2_O, then freeze-dried twice. Spectra were recorded in D_2_O at 25 °C on a Bruker Avance 500 MHz or on a Bruker Avance 600 MHz spectrometer (Karlsruhe, Germany). Both instruments were equipped with 5-mm TCI cryoprobe. ^1^H mono-dimensional NMR spectra were acquired with 128 scans. Water pre-saturation was applied during each 12 s of relaxation delay. HSQC spectra were obtained in phase-sensitive, sensitivity pure-absorption mode with decoupling in the acquisition period (Bruker pulse program hsqcetgpsisp.2). Processing of data was made using standard Bruker TOPSPIN 3.0 software (Karlsruhe, Germany).

### 4.7. Enzymatic Cleavage of HS

GAG mixtures from cartilage samples underwent a double enzymatic treatment with a mixture of heparin lyases I, II, and III (Grampian Enzymes, 3 mU each for 0.1 mg of starting material, according to USP-NF procedure *<207>1,6-Anhydro Derivative for Enoxaparin Sodium*), in 100 mM sodium acetate buffer, pH 7, and 10 mM calcium acetate. The reaction was stirred at 37 °C (Termo shaker TS-100 Biosan, Riga, Latvia) for 48 h, then stopped by boiling for 10 min followed by 0.2-μm filtration (LabService Analytica, Anzola dell’Emilia, Bologna, Italy).

### 4.8. Isolation of Digestion Products

Digested samples were loaded onto 3-kDa (fractions B) or 10-kDa (fractions A) MWCO Amicon Ultra Centrifugal Devices (Millipore, St. Louis, MO, USA) and recovered after 15 runs of centrifugation at 5000 rpm for 40 min (NuveNF200, Turkey). Permeates were freeze-dried, then dissolved in 200 μL of water and loaded onto a G10 desalting column (h 25 cm, Ø 1.2 cm) equilibrated in water and 10% ethanol (EtOH; Girelli, Italy) previously filtered and degassed. Digestion products were eluted at a flux speed of 0.7 mL/min and fractions of 30 s were collected and read at 210–232 nm (Cary50UV, Varian, Palo Alto, CA, USA). Recovered fractions containing the digestion products were freeze-dried and the desalting step was repeated to allow a better separation between salt and oligosaccharides.

### 4.9. IPRP-HPLC/ESI-TOF-MS Analysis

HPLC–MS analysis was performed on an LC system (Dionex Ultimate 3000, Dionex, Sunnyvale, CA, USA) equipped with degassing system (model LPG-3400), pump (model LPG-3400A), autosampler (model WPS-3000TSL), and ultraviolet (UV) detector (model VWD-3100) and coupled with an ESI-Q-TOF mass spectrometer (micrOTOF_Q_, Bruker Daltonics, Bremen, Germany). The chromatographic separation was performed using a Kinetex C18 analytical column (100 × 2.1 mm inner diameter, 2.6 μm particle size, Phenomenex, Torrance, CA, USA) with Security Guard Cartridges Gemini C18 (4 × 2.0 mm, Phenomenex). A binary solvent system was used for gradient elution. Solvent A (10 mM DBA, 10 mM CH_3_COOH in water or water/methanol 90:10) and solvent B (10 mM DBA and 10 mM CH_3_COOH in methanol) were delivered at 0.1 mL/min. Oligosaccharides were separated using a multi-step gradient as reported in the Table 3; fetal samples were eluted with a shorter and faster gradient, then different conditions were adjusted for adolescent and prepubescent samples. For this reason, small shifts in the elution time of oligosaccharides can be observed in LC chromatograms. The solvent composition was held for the last 19 min for equilibrating the chromatographic column before the injection of the next sample.

The MS spectrometric conditions were as follows: ESI in negative ion mode, drying gas temperature +180 °C, drying gas flow rate 7.0 L/min, nebulizer pressure 0.9 bar, and capillary voltage +3.2 kV. Sample ionization was obtained using optimized MS conditions of spray voltage and capillary temperature (3166 V and 350 °C, respectively). The mass spectra of the oligosaccharides were acquired in scan mode (*m*/*z* scan range 200–2000). Calibration of the mass spectrometer was obtained using an ES tuning mix solution acetonitrile solution (Agilent Technologies, Santa Clara, CA, USA) according to a standard procedure. Data were processed using the DataAnalysis software (HyStar Compass, version 3.0, Bruker Daltonics, Bremen, Germany). MS/MS fragmentation experiments were performed by collision induced fragmentation (CID) using the collision energy optimized to generate the appropriate fragment-rich mass spectrum.

### 4.10. Isolation of GAGs from Aggrecan

Aggrecan (1 mg, Sigma Aldrich) was dissolved in phosphate-buffered saline (PBS), 1 mM CaCl_2_, pH 7.4, and digested with 70 µg of Proteinase K at 50 °C for 48 h followed by heat inactivation and filtration with 0.22-µm cut-off. GAGs were recovered by ultrafiltration with 3-kDa cut-off and freeze-dried in D_2_O for NMR analysis.

## 5. Conclusions

The first important results of the present study were the isolation of HS from human growth plate cartilage, and determining its relative abundance in multiple osteochondromas and in peripheral chondrosarcomas compared to healthy conditions. Moreover, the analytical procedure applied pointed out two macroscopic aspects of HS structure, i.e., the degree of sulfation and chain length, which appeared to be significantly different in OC and CS with respect to healthy cartilage. Interestingly, it seems that HS structural analysis could be predictive of a possible malignant degeneration of benign tumor.

## Figures and Tables

**Table 1:** List of healthy and pathologic samples. Information on patients (gender, and age at resection), type of mutation, and area of excision are reported. Amount of starting wet cartilage: about 100 mg for prepubescent growth plate (GRP), and 150–200 mg for all other samples. FT—fetal growth plate; OC—osteochondromas; CS—chondrosarcomas; nd—not detected; EXT—exostosin gene; F—female; M—male.

Sample	Type of Mutation	Nucleotide Change	Area of Excision	Gender	Age ^▲^
**Healthy Cartilage**					
FT1	nd	-	-	M	19 w
FT2	nd	-	-	M	17 w
FT3	nd	-	-	M	17 w
FT4	nd	-	-	F	35 w
GRP1	None	-	Femur	M	10
GRP2	None	-	Femur	F	7
**Pathologic Cartilage**					
OC1	*EXT1* exon 7, splice site	c.1633-1G > C	Right distal femur	F	15
OC2	*EXT1* exon 2, missense	c.1019G > A(p.Arg340His)	Tibia	M	15
OC3	*EXT1* exon 2, missense	c.1019G > A(p.Arg340His)	Left proximal fibula	M	11
OC4	*EXT1* in toto deletion	-	Right proximal homer	M	14
OC5	*EXT2* exon 4, frameshift	c.669_670insG (p.Gln224Alafs*11)	Femur	M	16
OC6 ^■^	*EXT2* exon 8, nonsense	c.514C > T (p.Gln172*)	Right chest	M	5
OC7 ^■^	*EXT2* exon 8, nonsense	c.514C > T (p.Gln172*)	Right chest	M	5
OC8	*EXT2* exon 8, frameshift	c.834del (p.Glu278Aspfs*4)	Right distal femur	M	29
CS1 §	*EXT1* exon 10, nonsense	c.2038G > T (p.Glu680*)	Pubis	F	36
CS2 §	*EXT1* exon 10, nonsense	c.2038G > T (p.Glu680*)	Pubis	F	36
CS3	*EXT2* exon 2, nonsense	c.67C > T (p.Arg23*)	Right ileum	M	25
CS4	*EXT1* exon 10, nonsense	c.1165-3C > G	Right iliac wing	F	22
CS5 $	*EXT1* exon 1 deletion	-	Fibula	F	21
CS6 $	*EXT1* exon 1 deletion	-	Fibula	F	21

^▲^ Age is expressed in years unless otherwise indicated (w = weeks). ^■^ OC6 and OC7: excisions from the same patient and area. § CS1 and CS2: excisions from the same patient and tumor. $ CS5 and CS6: excisions from the same patient, different areas of the same lesion, with CS6, in particular, being from perichondrium.

**Table 2:** Summary table of the quantitative evaluation of HPLC–MS profiles of all the analyzed samples. Fr.A/Fr.B represents the ratio between the total area of peaks of the corresponding fractions A and B. %ΔU2,3,0 expresses the percentage value of the trisulfated disaccharide peak area with respect to the total peak area, specifically the percentage mean value of A and B fractions. The type of mutation is reported again as in Table 1.

Sample	Type of Mutation	Fr.A/Fr.B	%ΔU2,3,0
**Healthy Cartilage**			
FT1	nd	na	2.5
FT2	nd	na	1.8
FT3	nd	na	nd
FT4	nd	na	nd
GRP1	None	0.1	nd
GRP2	None	1.0	9
**Pathologic Cartilage**			
OC1	*EXT1* exon 7, splice site	0.9	58
OC2	*EXT1* exon 2, missense	0.4	12
OC3	*EXT1* exon 2, missense	<0.1	43
OC4	*EXT1* in toto deletion	0.4	48
OC5	*EXT2* exon 4, frameshift	0.1	10
OC6	*EXT2* exon 8, nonsense	0.2	20
OC7	*EXT2* exon 8, nonsense	0.1	65
OC8	*EXT2* exon 8, frameshift	3.1	45
CS1	*EXT1* exon 10, nonsense	4.6	67
CS2	*EXT1* exon 10, nonsense	1.5	53
CS3	*EXT2* exon 2, nonsense	12	64
CS4	*EXT1* exon 10, nonsense	0.1	6
CS5	*EXT1* exon 1 deletion	6.3	58
CS6	*EXT1* exon 1 deletion	3.2	52

nd: not detected; na: not applicable.

**Table 3:** Multi-step gradient elution.

Solvent A	Solvent B	Gradient (% B)		Injection (µL)
100% H_2_O	100% MeOH	t = 0	10	30 of 100
		t = 40	35	
		t = 85	50	
		t = 88	90	
		t = 95	10	
H_2_O/MeOH 90:10	100% MeOH	t = 0	10	30 of 100
		t = 60	40	
		t = 65	90	
		t = 75	10	

## Supplementary Materials

The Supplementary Materials are available online.
