# Supplementary material for: Structural Features of Heparan Sulfate from Multiple Osteochondromas and Chondrosarcomas
Source: Molecules. 2018 Dec 11;23(12):3277. doi: 10.3390/molecules23123277 (PMC6321082; doi:10.3390/molecules23123277)
Supplement: Supplementary file 1 [file molecules-23-03277-s001.pdf]

Supplementary Materials

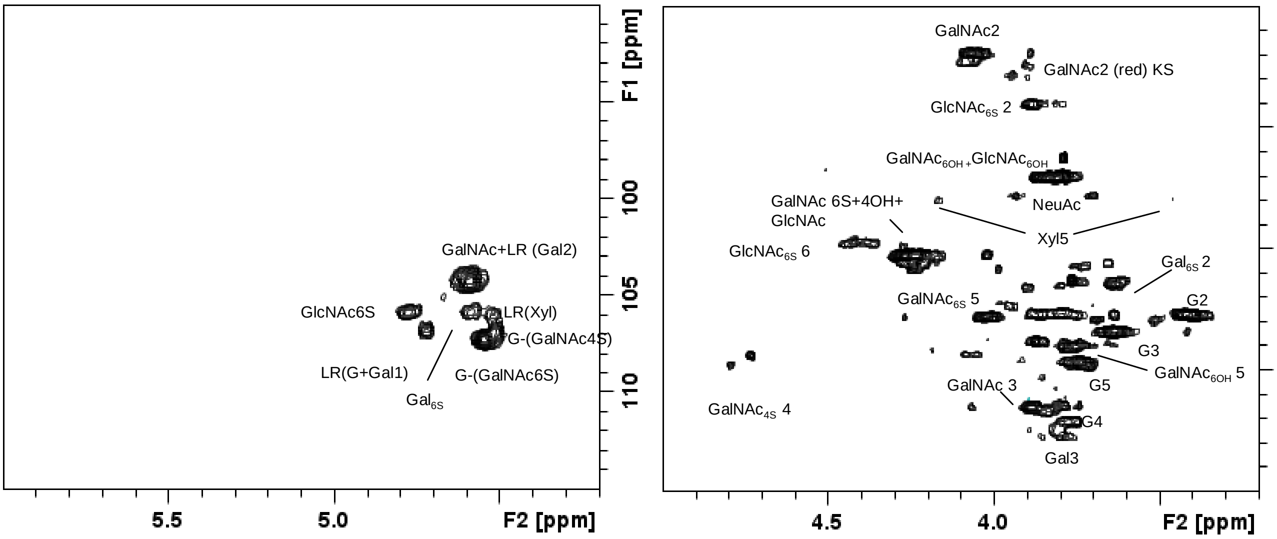

FIGURE S1. HSQC-NMR spectrum of GAGs from Aggrecan. Signals of the anomeric region are shown on the left while signals from the backbone are shown on the right. GalNAc: N-acetyl galactosamine, GlcNAc: N-acetyl-glucosamine, G: glucuronic acid, Gal: galactose, NeuAc: neuraminic acid, Xyl: xylose. CSs are constituted by GalNAc and G, while type II KS is constituted by Gal and GlcNAc units with NeuAc residues at the non-reducing end and GalNAc at the reducing end.

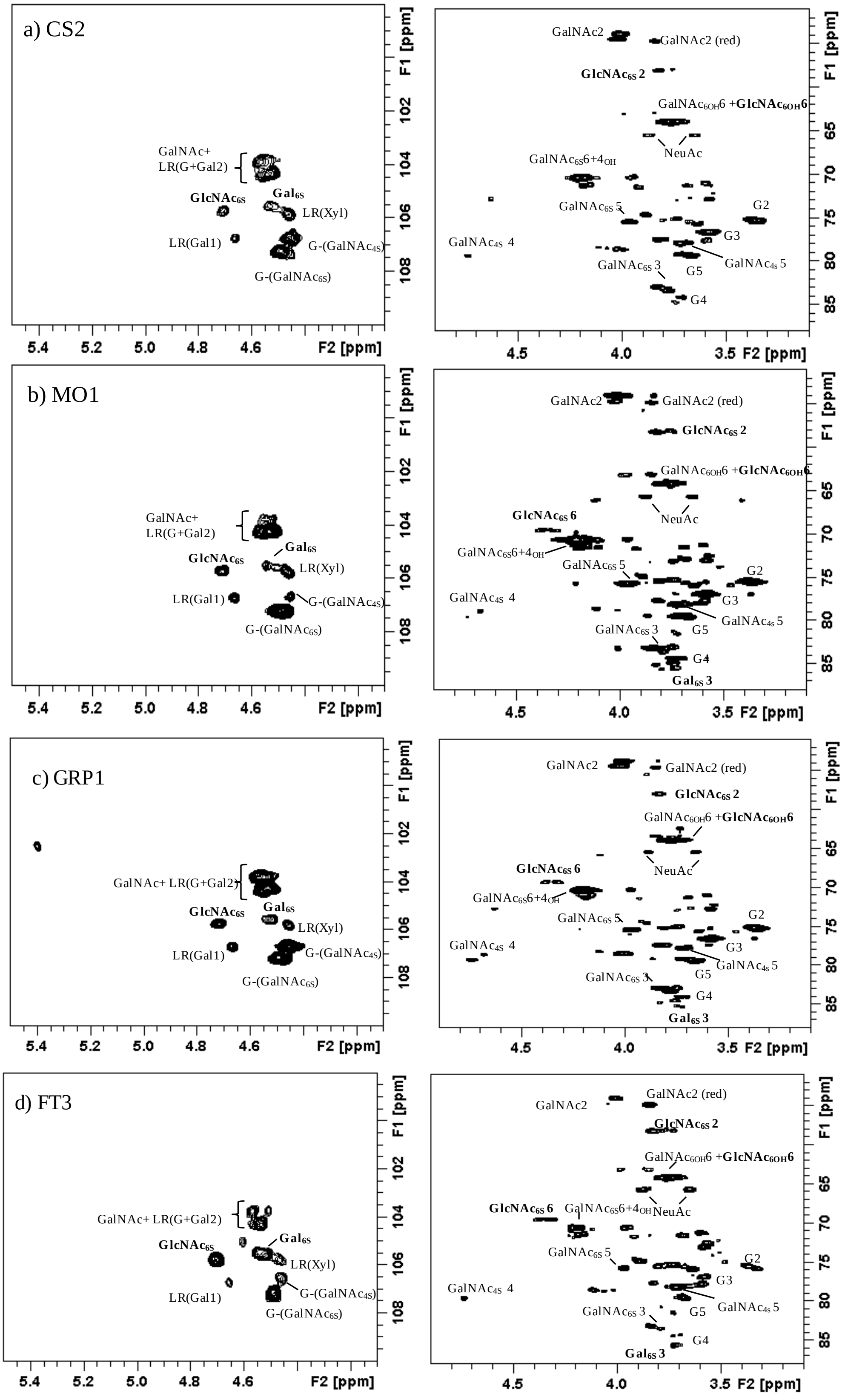

FIGURE S2. HSQC-NMR spectra of GAGs from cartilage: a) CS2, b) MO1, c) GRP1, d) FT3. Signals of the anomeric region are shown on the left while signals from the backbone are shown on the right. GalNAc: N-acetyl galactosamine, GlcNAc: N-acetyl-glucosamine, G: glucuronic acid, Gal: galactose, NeuAc: neuraminic acid, Xyl: xylose, LR: linkage region. Signal from KS are indicated in bold.

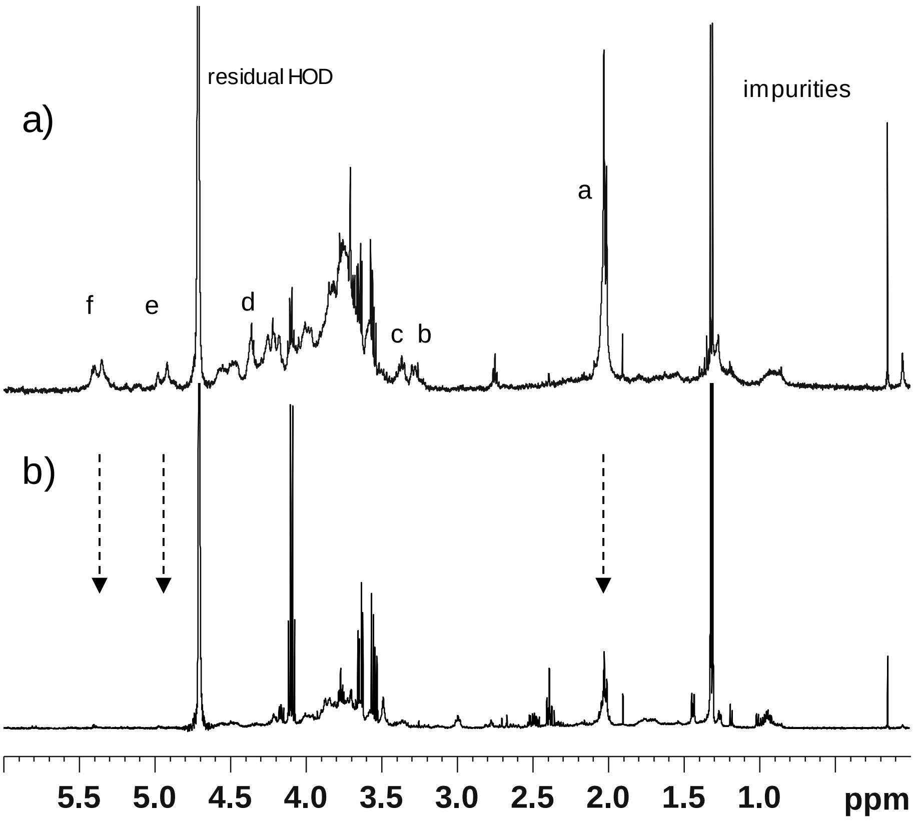

FIGURE S3. 1D-NMR spectra of CS5-B (< 10kDa) before, a) and after, b) digestion with heparinases. Signals of HS are indicated and correspond to: a. N-acetyl (CH_3) Glc-NAc (2.0 ppm); b. GlcNS, H-2 (3.2 ppm); c. GlcA, H-2 (3.4 ppm); e. IdoA, H-5 (4.9-5 ppm); f. GlcN (Ac or S) and IdoA2S H-1 (5.4 ppm). Arrows indicate the decrease of signals.

.

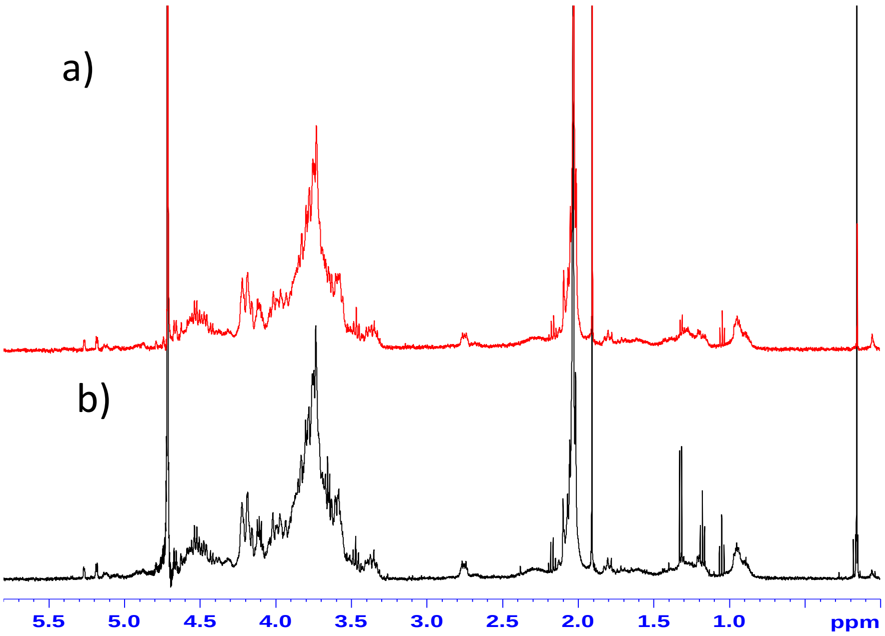

FIGURE S4. 1D-NMR spectra of GRP2-B before, a) and after, b) digestion with heparinases. No structural changes can be observed.

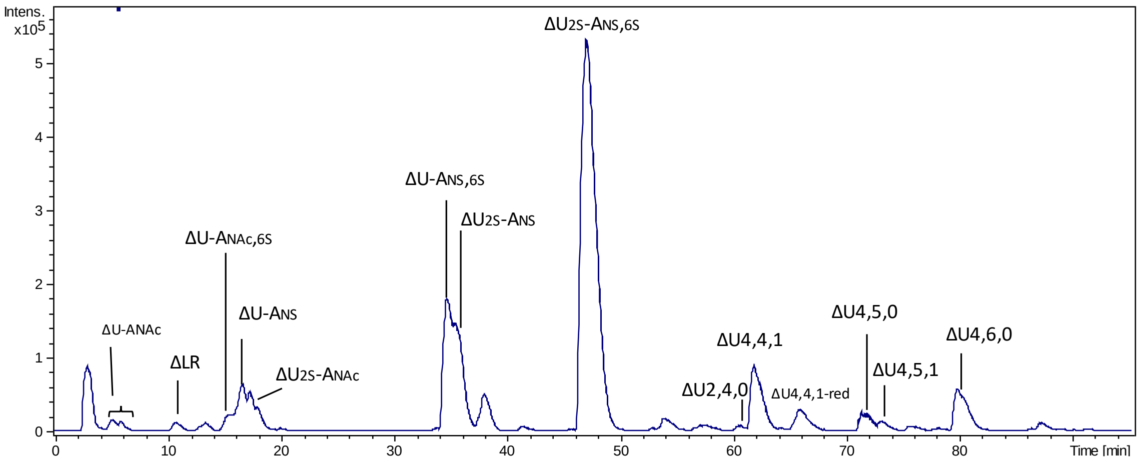

FIGURE S5. LC-MS profile of commercial porcine heparin digested with heparinases I, II, III mixture: structure assignment of the main peaks.

TABLE S1. Experimental data obtained by MS/MS fragmentation of the ion at m/z 774.148, attributed to ΔU4,2,2-LR. The selected ion was fragmentated with a collision energy of 50 eV and obtained ions are reported.

Experimental m/z	z	Mass assignment	Theoretical m/z	Error (ppm)
157.0135	-1	ΔU (-H_2O)	157.0132	1.9
161.0452	-1	Gal (-H_2O)	161.0445	4.3
175.0244	-1	ΔU	175.0237	4.0
193.0347	-1	U	193.0343	2.1
282.0294	-1	$A_{NAc,6S}$ (-H_2O)	282.0278	5.7
300.0392	-1	$A_{NAc,6S}$	300.0384	2.7
316.5431	-2	ΔU3,1,1	316.5418	4.1
337.0758	-1	*ΔU-Gal*	337.0765	2.1
378.1050	-2	ΔU2,0,1	378.1031	5.0
396.1150	-1	U2,0,1	396.1137	3.3
458.0606	-2	ΔU2,1,1	458.0599	1.5
536.1259	-1	ΔU3,0,1 (-H_2O)	536.1246	2.4
616.0816	-1	ΔU3,1,1 (-H_2O)	616.0814	0.3
631.1705	-1	*ΔU*-Gal-Gal-Xyl	631.1716	1.7
649.1818	-1	*U-Gal-Gal-Xyl*	649.1822	0.6

TABLE S2. Experimental data obtained by MS/MS fragmentation of the ion at m/z 546.0, attributed to ΔU5,2,2. The selected ion was fragmentated with a collision energy of 50 eV and obtained ions are reported.

Experimental m/z	z	Mass assignment	Theoretical m/z	Error (ppm)
157.0144	-1	ΔU (-H_2O)	157.0132	7.6
175.0244	-1	ΔU	175.0237	4.0
282.0294	-1	$A_{NAc,6S}$ (-H_2O)	282.0278	5.7
300.0378	-1	$A_{NAc,6S}$	300.0384	2.0
396.1150	-1	U2,0,1	396.1137	3.3
458.0606	-2	ΔU2,1,1	458.0599	1.5
536.1187	-1	ΔU3,0,1 (-H_2O)	536.1246	11
554.1377	-1	ΔU3,0,1	554.1363	2.5

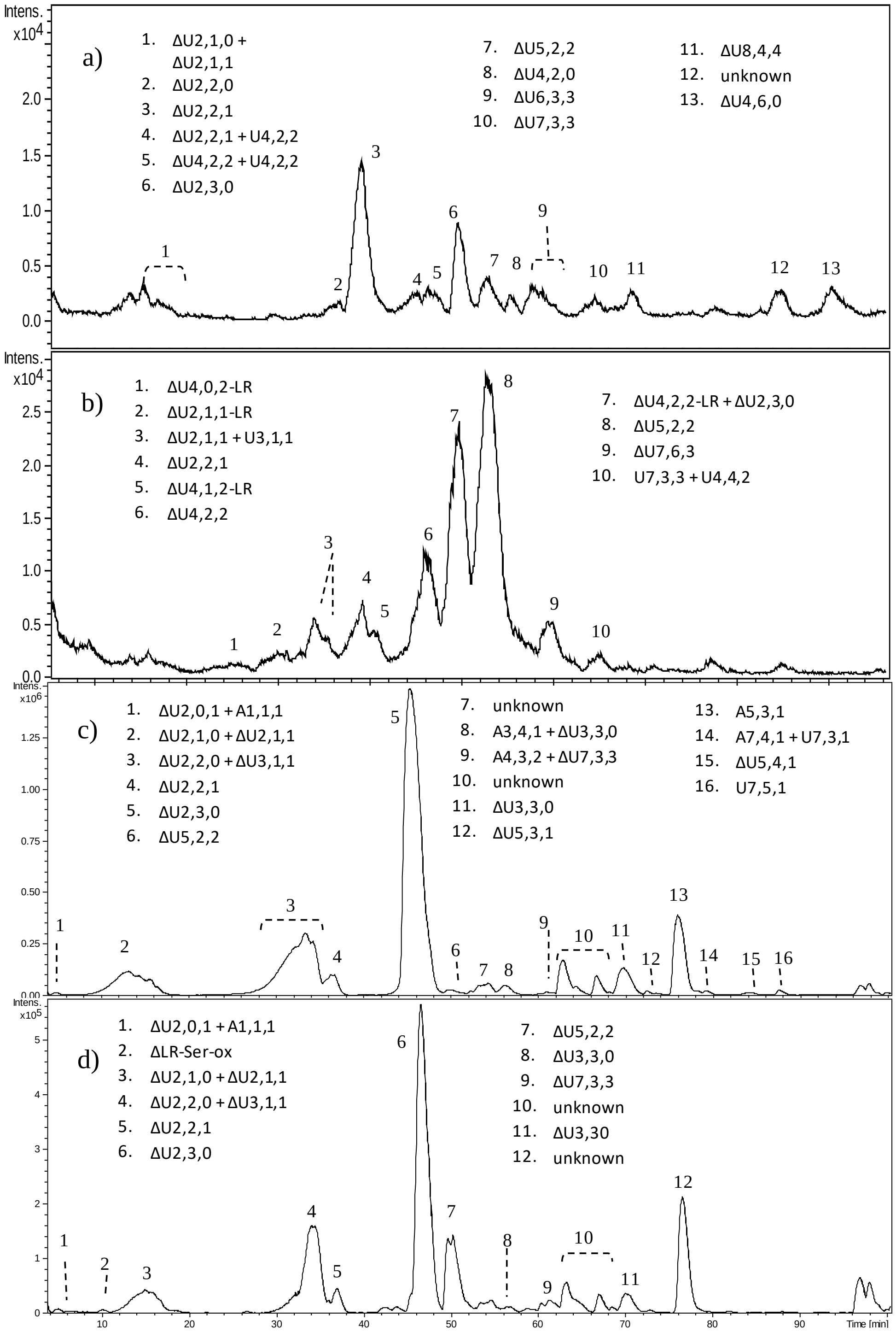

FIGURE S6. LC-MS profiles of heparinases digestion of HS from osteochondromas. a) OC2-A >10 kDa, b) OC2-B <10 kDa, c) OC8-A >10 kDa, d) OC8-B <10 kDa.

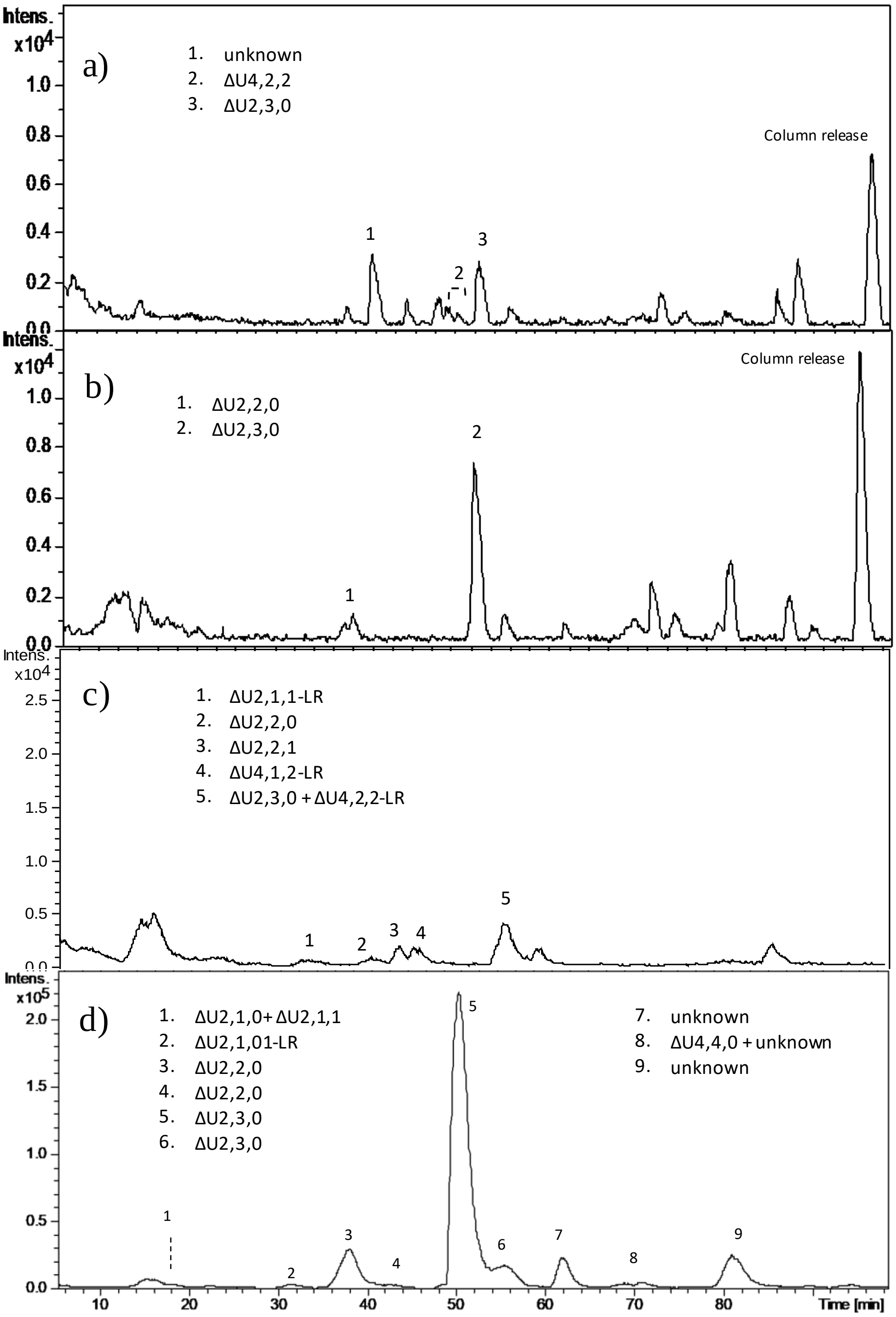

FIGURE S7. LC-MS profiles of heparinases digestion of HS from osteochondromas. a) OC1-A >10 kDa, b) OC1-B <10 kDa, c) OC3-A >10 kDa, d) OC3-B <10 kDa.

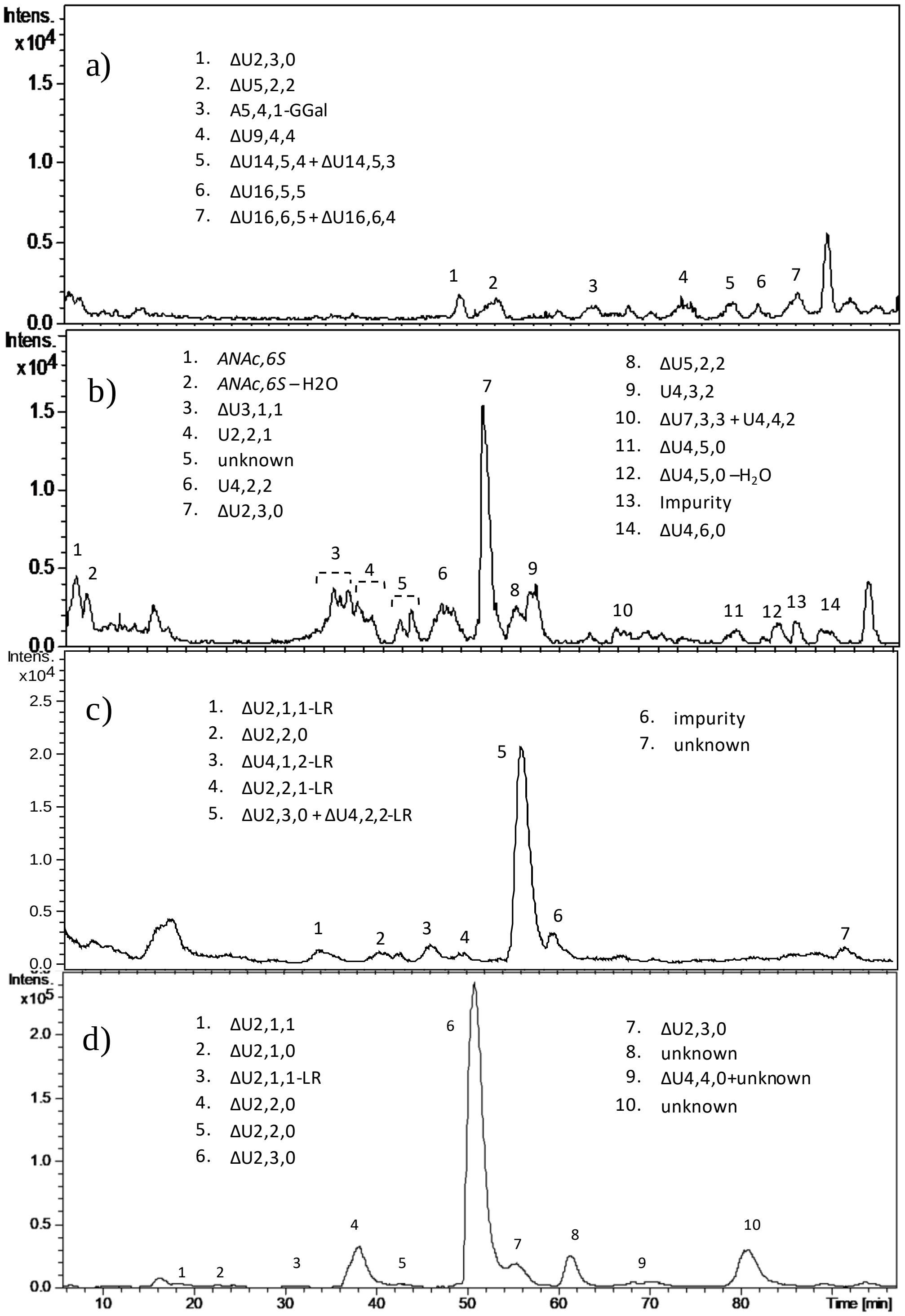

FIGURE S8. LC-MS profiles of heparinases digestion of HS from osteochondromas. a) OC6-A >10 kDa, b) OC6-B <10 kDa, c) OC7-A >10 kDa, d) OC7-B <10 kDa.

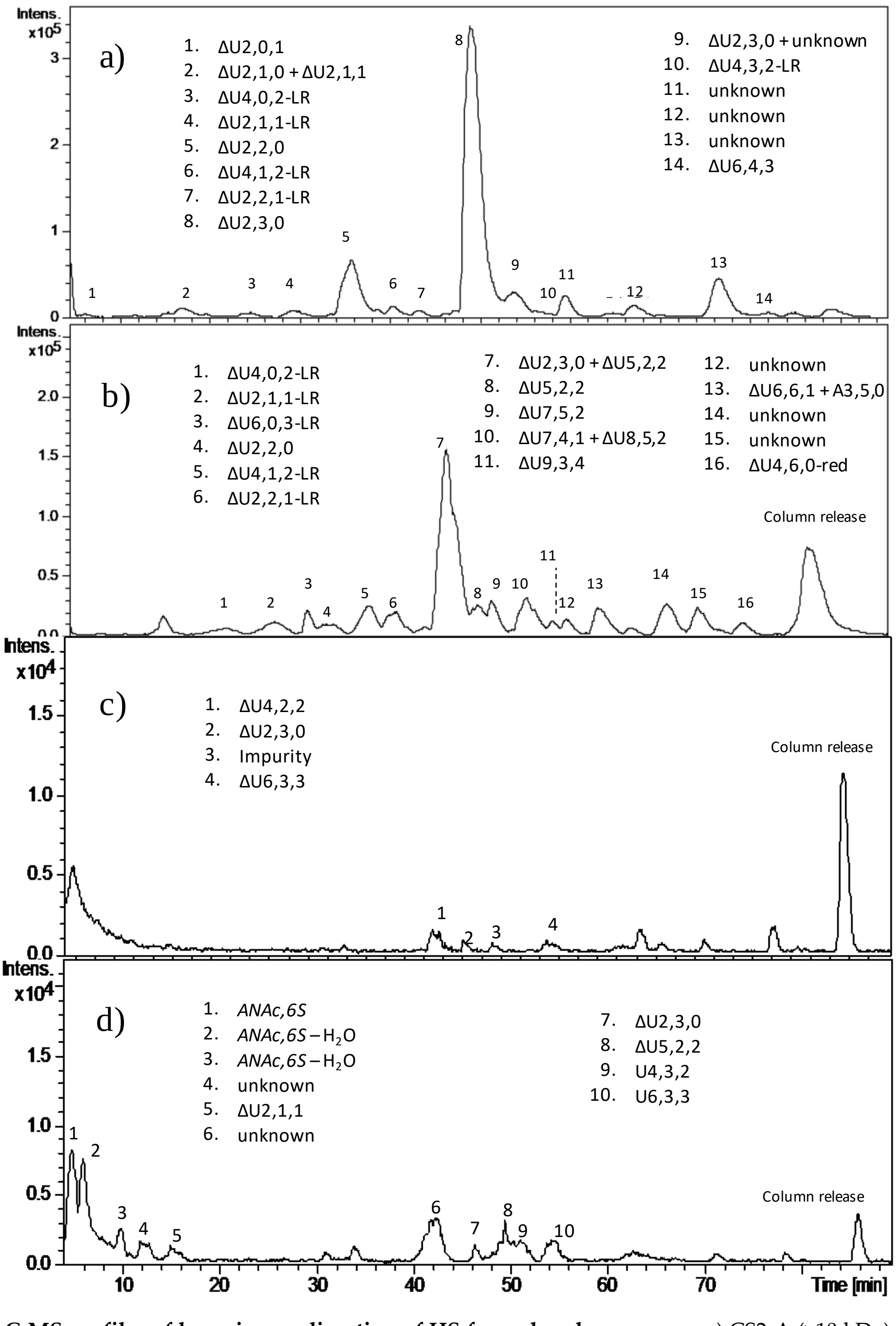

FIGURE S9. LC-MS profiles of heparinases digestion of HS from chondrosarcomas. a) CS2-A (>10 kDa) (b) CS2-B (<10 kDa), c) CS4-A (>10 kDa), d) CS4-B (<10 kDa).

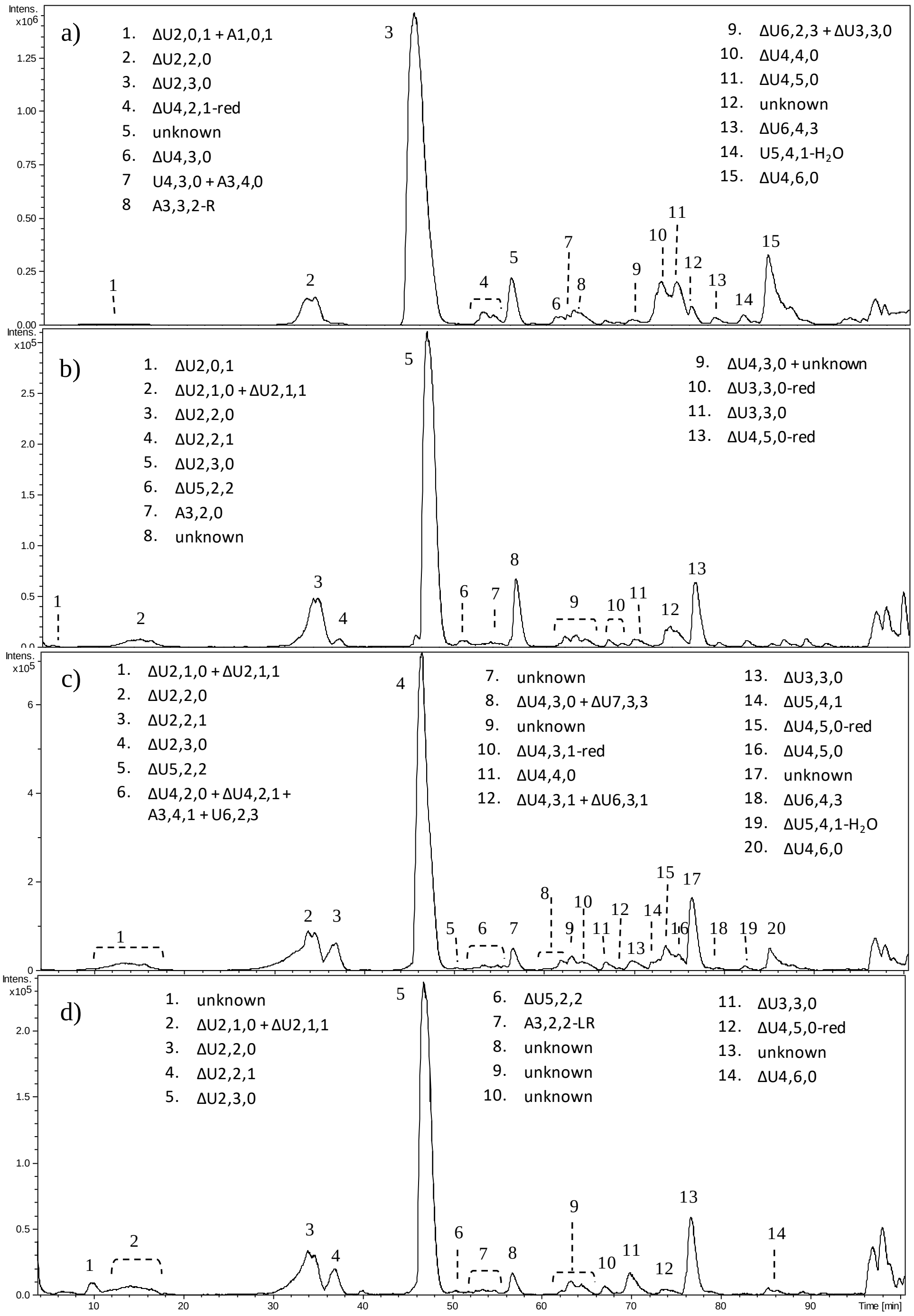

FIGURE S10. LC-MS profiles of heparinases digestion of HS from chondrosarcomas. a) CS5-A (>10 kDa) (b) CS5-B (<10 kDa), c) CS6-A (>10 kDa), d) CS6-B (<10 kDa).
